# Comparative effects of kinect-based versus therapist-based constraint-induced movement therapy on motor control and daily motor function in children with unilateral cerebral palsy: a randomized control trial

**DOI:** 10.1186/s12984-023-01135-6

**Published:** 2023-01-27

**Authors:** Tsai-Yu Shih, Tien-Ni Wang, Jeng-Yi Shieh, Szu-Yu Lin, Shanq-Jang Ruan, Hsien-Hui Tang, Hao-Ling Chen

**Affiliations:** 1 School of Occupational Therapy, College of Medicine, National Taiwan University, Taipei, Taiwan; 2 Department of Physical Medicine and Rehabilitation, National Taiwan University Hospital, Taipei, Taiwan; 3 Department of Electronic and Computer Engineering, National Taiwan University of Science and Technology, Taipei, Taiwan; 4 Department of Design, National Taiwan University of Science and Technology, Taipei, Taiwan

**Keywords:** Cerebral palsy, Children, Constraint-induced therapy, Virtual reality, Upper extremity

## Abstract

**Background:**

Constraint-induced movement therapy (CIMT) is a prominent neurorehabilitation approach for improving affected upper extremity motor function in children with unilateral cerebral palsy (UCP). However, the restraint of the less-affected upper extremity and intensive training protocol during CIMT may decrease children’s motivation and increase the therapist’s workload and family’s burden. A kinect-based CIMT program, aiming to mitigate the concerns of CIMT, has been developed. The preliminary results demonstrated that this program was child-friendly and feasible for improving upper extremity motor function. However, whether the kinect-based CIMT can achieve better or at least comparable effects to that of traditional CIMT (i.e., therapist-based CIMT) should be further investigated. Therefore, this study aimed to compare the effects of kinect-based CIMT with that of therapist-based CIMT on upper extremity and trunk motor control and on daily motor function in children with UCP.

**Methods:**

Twenty-nine children with UCP were recruited and randomly allocated to kinect-based CIMT (*n* = 14) or therapist-based CIMT (*n* = 15). The intervention dosage was 2.25 h a day, 2 days a week for 8 weeks. Outcome measures, namely upper extremity and trunk motor control and daily motor function, were evaluated before and after 36-h interventions. Upper extremity and trunk motor control were assessed with unimanual reach-to-grasp kinematics, and daily motor function was evaluated with the Revised Pediatric Motor Activity Log. Between-group comparisons of effectiveness on all outcome measures were analyzed by analysis of covariance (α = 0.05).

**Results:**

The two groups demonstrated similar improvements in upper extremity motor control and daily motor function. In addition, the kinect-based CIMT group demonstrated greater improvements in trunk motor control than the therapist-based CIMT group did (F(1,28) > 4.862, p < 0.036).

**Conclusion:**

Kinect-based CIMT has effects comparable to that of therapist-based CIMT on UE motor control and daily motor function. Moreover, kinect-based CIMT helps decrease trunk compensation during reaching in children with UCP. Therefore, kinect-based CIMT can be used as an alternative approach to therapist-based CIMT.

*Trial registration:* ClinicalTrials.gov Identifier: NCT02808195. Registered on 2016/06/21, https://clinicaltrials.gov/ct2/show/NCT02808195.

## Background

Motor impairment of an upper extremity (UE) is a major deficit in children with unilateral cerebral palsy (UCP). This deficit may further limit children’s participation in daily activities, education and play [1]. The tendency of children with UCP to avoid using their affected UE due to unsuccessful sensorimotor experiences is known as developmental disregard (DD) [2]. This phenomenon has been found to decrease the frequency and quality of using the affected UE, even if the affected UE has sufficient ability to perform tasks. Children with UCP usually perform bimanual activities with only the less-affected UE [3]. If the affected UE must be used, greater trunk movement is used to compensate for the decreased function of the affected UE [4]. The nonuse or poor use of the affected UE may cause muscle weakness, joint contracture and asymmetrical movement development [5]. Therefore, interventions for the motor function of the affected UE in children with UCP have been developed to overcome the DD phenomenon.

Constraint-induced movement therapy (CIMT) is a prominent neurorehabilitation approach for improving affected UE motor function in children with UCP. The two main features of CIMT are restraint of the less-affected UE and intensive structured training of the affected UE. The restraint of the less-affected UE helps to force the use of the affected UE. The massive and repetitive practice of the affected UE movements with a shaping strategy helps improve motor function and reverse the DD of the affected UE [6]. Several systematic reviews have proven that CIMT can effectively improve motor function of the affected UE, with medium to large effects [2, 6]. However, some practical issues should be considered when applying CIMT. The restraint of the less-affected UE during CIMT may induce children’s frustration and refusal to participate [7]. In addition, the intensive training protocol may raise some concerns. First, repetitive tasks used in intensive training may limit the motivation of the children with UCP. Second, an intensive CIMT protocol may place considerable time and other burdens on the families [7, 8]. Third, CIMT delivered by therapists may increase the labor burden, resulting in huge health care costs [9].

To facilitate the delivery of CIMT in clinical practice, the restraint type of the less-affected UE and the training protocols have been modified [6, 10, 11]. Some studies have selected less invasive methods to apply restraint, such as a short splint or mitten [11]. However, children can easily remove the restraint, which may reduce the effects of restraint [11]. In addition, to reduce therapists’ load in implementing intensive training for CIMT, the caregiver-directed CIMT model was developed [12–14]. However, caregiver-directed CIMT was found to further increase parents’ stress and family burdens [12]. Moreover, implementing CIMT is difficult for most parents even with weekly supervision by therapists. Only 60% of the targeted dosage is achieved in caregiver-directed CIMT [12, 13]. It seems that practical issues with CIMT still remain. A new modified CIMT protocol should be developed to increase the children-friendliness and feasibility of the original CIMT.

Virtual reality (VR), which allows intensive repetition of task practice in highly motivational games, has been recently explored as an alternative approach for motor rehabilitation in children with UCP [15]. In most of the studies, motor rehabilitation using a VR system has been found to improve the motivation of children with UCP to participate in motor training programs [16]. However, the effects on motor performance in children with UCP remain controversial, possibly due to varied intervention protocols, different treatment dosages and diverse VR systems [15]. In contrast, the intervention protocol and minimum dosage of CIMT to achieve UE motor improvement have been well-developed [6]. VR-based motor rehabilitation can provide repetitive, intensive tasks with multisensory feedback, allowing the delivery of the intensive motor training protocol of CIMT without decreasing children’s motivation or increasing the parents’ burden and therapist’s workload. Moreover, motor-detection sensors in a VR game design may help embed the restraint into a playful context, which may be an effective way to increase the cooperation of children with UCP when their less-affect UE is restrained for CIMT. It seems that VR can mitigate the disadvantages of CIMT, so it potentially can be used as a mediator for implementing CIMT for children with UCP.

Combining the advantages of VR intervention and CIMT, a kinect-based CIMT program for children with UCP has been developed. In this program, the VR game was developed based on the principals of CIMT. The preliminary results demonstrated that this program was child-friendly and feasible for improving UE motor function [17]. However, whether the kinect-based CIMT can achieve better or at least comparable effects to that of traditional CIMT (i.e., therapist-based CIMT) should be further investigated so as to improve the clinical utility of CIMT. Therefore, the purpose of the present study was to compare the effects of kinect-based CIMT and that of therapist-based CIMT on UE and trunk motor control and on daily motor function.

## Methods

### Procedure

This study was a single blind randomized controlled trial. All participants were randomly assigned to either the kinect-based CIMT or therapist-based CIMT group. The group allocation was concealed and randomized using a computer-generated list of random sequence. Both groups received 36-h interventions by licensed occupational therapists in the children’s natural environments, such as their homes or schools, for 2.25 h a day, 2 days a week for 8 weeks. This study was approved by the Research Ethics Committee of National Taiwan University Hospital (No. 201601057RINB) and registered at ClinicalTrials.gov (NCT02808195). All participants and their parents provided informed consent before participating in the study.

### Participants

The participants were recruited from cerebral palsy associations, medical centers and special education systems. Inclusion criteria were: (1) aged 5 to 12 years; (2) diagnosis of congenital UCP; (3) considerable nonuse of the affected UE (amount-of-use score of the Revised Pediatric Motor Activity Log (PMAL-R) < 2); (4) no excessive muscle tone (Modified Ashworth Scale ≤ 2 at any joint of the UE); (5) no severe cognitive, visual, or auditory disorders according to medical documents, parental reports, and the examiner’s clinical observation during the baseline evaluation; (6) no botulinum toxin A injection or operations on the less-affected UE within 6 months.

### Interventions

For the kinect-based CIMT group, the treatment was provided by our kinect-based CIMT games, called “Adventure Island” and “Kitten Island” [17]. Both games were run on a laptop running Windows 8, to which the Kinect 2 sensor was connected. The kinect-based CIMT intervention was performed in the children’s natural environments (e.g., homes, schools) and supervised by therapists. During each intervention session, each game was played for at least 1 h, playing these 2 games took 2.25 h in total. Since difficulties in shoulder flexion, elbow extension, forearm supination, and voluntary movement of the fingers of the affected side have been revealed in children with UCP, the categories of arm-reaching, manipulation, and arm-hand tasks were designed in our games. [18–20]. In “Adventure Island”, the child acted as a warrior, collecting cannonballs and defeating monsters. The training movements were reaching, grasping, releasing, holding and aiming with the affected UE. In “Kitten Island”, the child was asked to catch fish and pick apples for the kitten. The training movements were tracking, flapping and forearm supination/pronation. To provide intensive structured training, the task difficulties in the temporal and spatial dimensions and the accuracy of movement in the games could be adjusted by therapists through a therapist’s interface. If a 10% improvement in the performance score of the game was achieved, the task difficulties in terms of task repetitions, reaching distance or accuracy of required movements were increased [17]. To restrain the less-affected UE, contextual restraint was applied. The kinect-based CIMT program provides a playful restraint context wherein the players must put their less-affected hand on one knee so that the warriors in the game can stabilize a box or protect themselves from a monster’s attacks. Moreover, a pause mechanism during gameplay prevented inadequate compensatory movements (e.g., excessive trunk movement) and ensured the player’s safety. If the children exhibited excessive trunk flexion or deviation, the game paused and a warning appeared on the screen to instruct the user to assume the correct position. To increase the child’s adherence to the intervention, a leaderboard and a badge collection book were designed in kinect-based CIMT program. A detailed description of this kinect-based CIMT intervention can be found in our previous study [17].

For the therapist-based CIMT group, the intervention was provided according to personal goals and preferences. The intervention was mainly delivered by the therapist and the family members were encouraged to assist and participate in the intervention. As in the kinect-based CIMT group, training program in the therapist-based CIMT group were the categories of arm-reaching, manipulation, and arm-hand tasks. During each intervention session, 2 to 3 age-appropriate therapeutic games, such as board games, crafts, and manipulation activities, were chosen based on the CIMT principles and the child’s interests. To provide intensive structured training, the difficulties of the therapeutic games were graded by the therapists according to each child’s ability and progression. Appropriate feedback was also given by the therapists to enhance motor learning. To restrain the less-affected UE, the gentle restraint methods of applied verbal instruction and gentle physical guidance were used. A detailed description of this friendly-CIT intervention can be found in our previous study [10].

### Outcome measures

Outcome measures, namely UE and trunk motor control and daily motor function, were evaluated before and after 36-h interventions. All participants were assessed by a certified occupational therapist who was unaware of the group to which the participant had been allocated.

The unimanual reach-to-grasp task was used to assess UE and trunk motor control. During the experiment, the participants were instructed to reach for and grasp the pegs with the affected hand as fast and as accurately as possible after hearing a start signal. The peg was placed in front of the affected hand at 90% of the arm length, measured from the acromion to the midpoint of the radius and ulnar styloid process. The diameter and height of the peg were 3.2 and 6.6 cm, respectively. Five successful trials were recorded after practice.

Three-dimensional kinematic data were collected by a 6-camera motion capture system (Vicon MX, Oxford Metrics Group, U.K.) at a sampling rate of 100 Hz. Reflective markers were placed on the suprasternal notch, 7th cervical vertebrae, 8th thoracic vertebrae, xiphoid process, radius styloid process and ulnar styloid process of the affected wrist. The midpoint of the radial and ulnar styloid process was used to represent the endpoint. Movement onset and offset were defined as 5% of the peak velocity of the endpoint trajectory.

The PMAL-R, a parent-reported assessment, was used to assess the spontaneous use of the affected UE in 22 daily activities [21]. In this measure, amount of use and quality of use of the affected UE for the activities are rated on 6-point ordinal scales (0–5). A higher score indicates greater use frequency or better movement quality of the affected UE.

### Data reduction

The variables of UE motor control included reaction time (RT), movement time (MT), peak velocity (PV), percentage of MT when PV occurred (PPV), and movement unit (MU). PV, PPV and MU were calculated in the horizontal plane and vertical direction. RT was defined as the interval between the start signal and onset of reaching, representing preplanning efficiency. MT was defined as the interval between onset and offset of reaching, representing movement efficiency. PV was the maximal endpoint velocity during reaching, which is indicative of the magnitude of force production. The PPV indicated the motor preplanning ability. One MU was defined as one acceleration and one deceleration phase; fewer MUs indicates better movement smoothness.

To investigate the trunk motor control during reaching, normalized trunk displacement (nTD), normalized endpoint displacement (nED) and trunk contribution slope (TCS) both before PPV occurred (PPV_before_) and after PPV occurred (PPV_after_) were analysed in the anterior–posterior directions. nTD was computed from the displacement of the suprasternal notch marker, normalized for reaching distance. nED was computed from the displacement of the endpoint minus the displacement of the suprasternal notch marker, normalized for reaching distance. TCS was defined as the ratio of nED to nTD. Typically developing children tend to use their arms to reach for a target, while children with UCP may move their trunks to compensate for the poor reaching function of their affected UE [4]. Therefore, lower nED and TCS values and higher nTD may indicate more trunk compensation.

### Statistical analysis

All data were analysed in SPSS version 20.0 (IBM Corp., Armonk, NY, USA). For baseline demographic information, the continuous variables and categorical variables were compared by independent *t* test or ${\chi }^{2}$ test, as appropriate. Analysis of covariance was used to compare the treatment effects on the outcome measures between groups while baseline scores were controlled as covariates. The effect size was calculated as partial eta squared (${\eta }_{p}^{2}$). The large, medium and small effects were represented by ${\eta }_{p}^{2}$ values of 0.138, 0.058 and 0.01, respectively. The level of significance was set at 0.05.

## Results

**Fig. 1:**
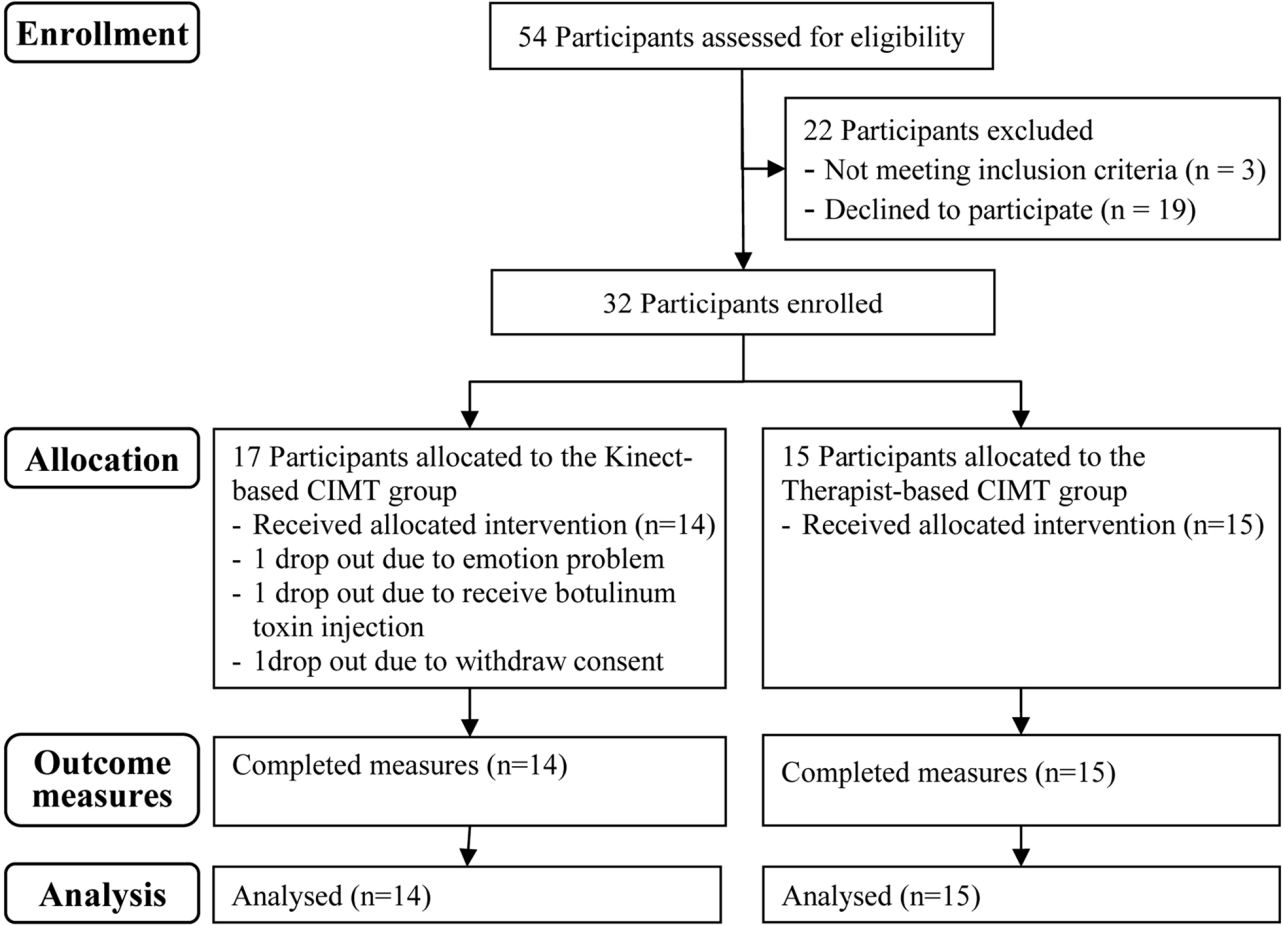
Consolidated Standards of Reporting Trials (CONSORT) flowchart. *CIMT* constraint-induced movement therapy

Form August 2016 to May 2021, thirty-two children with UCP were recruited. Three participants in the kinect-based CIMT group dropped out. In the end, a total of 29 children, 14 in the kinect-based CIMT group and 15 in the therapist-based CIMT group, completed all intervention sessions and the study procedure (Fig. 1). No significant differences in demographic characteristics were found between the groups (Table 1).

**Table 1:** Baseline participant characteristics

Characteristics	Kinect-based CIMT (*n* = 14)	Therapist-based CIMT (*n* = 15)	*p* value^a^
Age (years, mean ± SD)	8.487 (2.279)	8.279 (2.074)	0.798
Gender, n (%)			0.837
Male	8 (57.1)	8 (53.3)	
Female	6 (42.8)	7 (46.6)	
More affected hand, n (%)			0.876
Left	6 (42.8)	6 (40.0)	
Right	8 (57.1)	9 (60.0)	
MACS, n (%)			0.540
Level I	4 (28.5)	4 (26.6)	
Level II	6 (42.8)	9 (60.0)	
Level III	4 (28.5)	2 (13.3)	
Melbourne Assessment 2			
Range of motion (%, mean ± SD)	71.693 (18.295)	70.370 (22.174)	0.865
Accuracy (%, mean ± SD)	83.714 (20.242)	83.429 (22.890)	0.972
Dexterity (%, mean ± SD)	53.383 (22.316)	48.872 (26.390)	0.629
Fluency (%, mean ± SD)	63.946 (15.141)	64.625 (20.004)	0.920

^a^*p* values were calculated using an independent *t* test or${\chi }^{2}$ test. *SD* standard deviation, *CIMT* constraint-induced movement therapy, *MACS* Manual Ability Classification System

**Fig. 2:**
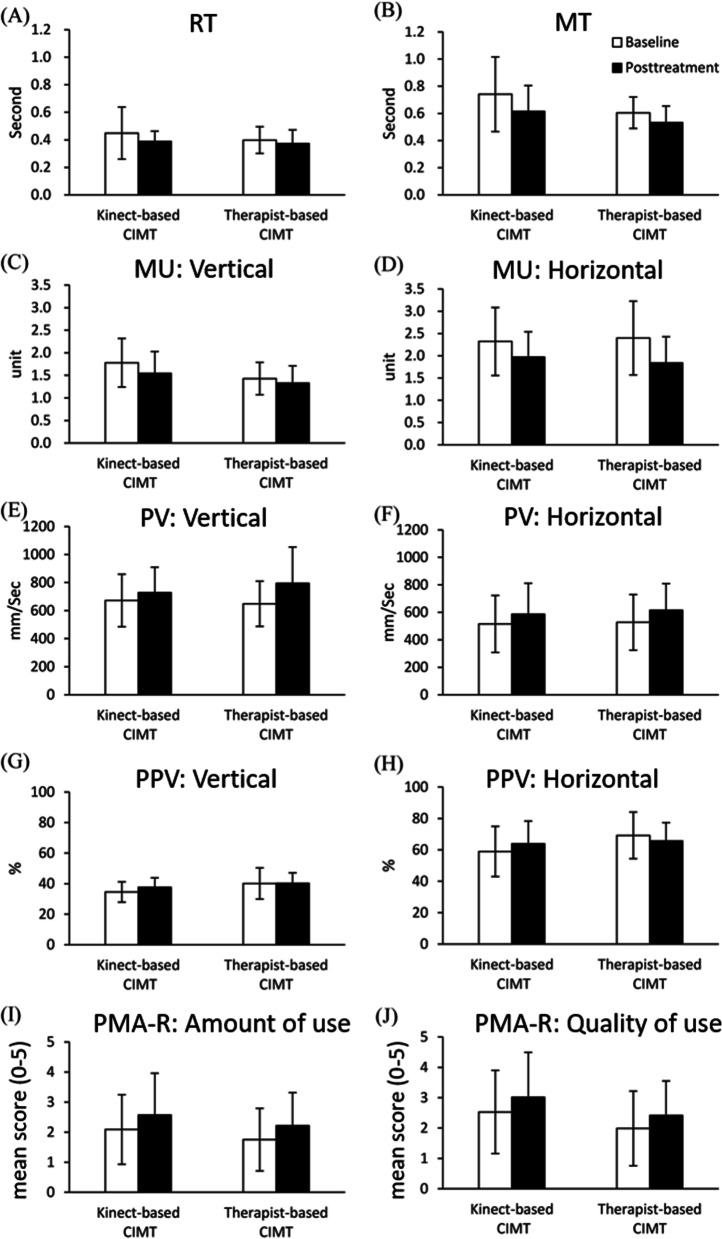
Between-group comparisons of upper extremity motor control and daily motor function analyzed by ANCOVAs using baseline scores as the covariate. For each outcome measure, the white bar represents the baseline score, and the black bar represents the posttreatment score. Whiskers represent standard deviations. *CIMT* constraint-induced movement therapy, *RT* reaction time, *MT* movement time, *MU* movement units, *PV* peak velocity, *PPV* percentage of MT when PV occurred, *PMAL-R* Revised Pediatric Motor Activity Log

**Fig. 3:**
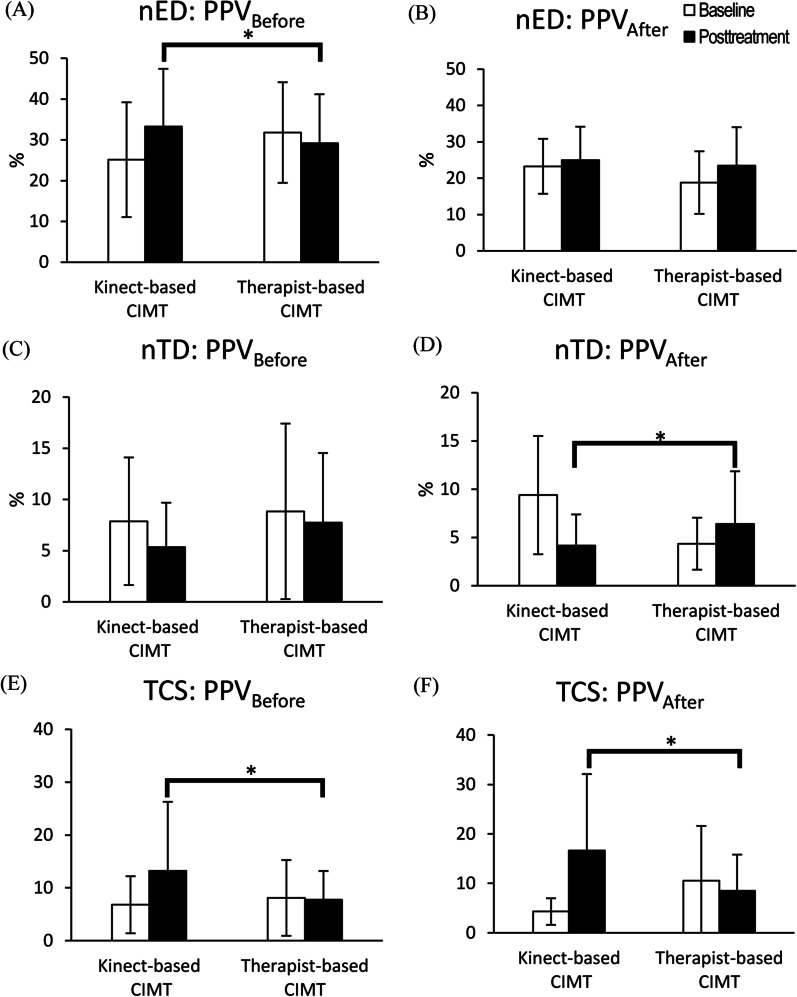
Between-group comparisons of trunk motor control analyzed by ANCOVAs using baseline scores as the covariate. For each outcome measure, the white bar represents the baseline score, and the black bar represents the posttreatment score. Whiskers represent standard deviations. Asterisks represent significant between-group posttreatment differences (p < 0.05). *CIMT* constraint-induced movement therapy, *PPV* percentage of movement time when peak velocity occurred, *nED* normalized endpoint displacement, *nTD* normalized trunk displacement, *TCS* Trunk contribution slope

For UE motor control, no significant between-group differences were found in RT (*F* (1, 28) = 0.031, *p* = 0.862, ${\eta }_{p}^{2}$ = 0.001), MT (*F* (1, 28) = 0.018, *p* = 0.895, ${\eta }_{p}^{2}$= 0.001), MU (Vertical: *F* (1, 28) = 0.032, *p* = 0.860, ${\eta }_{p}^{2}$ = 0.001; Horizontal: *F* (1, 28) = 0.706, *p* = 0.409, ${\eta }_{p}^{2}$ = 0.026), PV (Vertical: *F* (1, 28) = 0.031, *p* = 0.862, ${\eta }_{p}^{2}$ = 0.001; Horizontal: *F* (1, 28) = 0.150, *p* = 0.702, ${\eta }_{p}^{2}$ = 0.006) and PPV (Vertical: *F* (1, 28) = 0.447, *p* = 0.496, ${\eta }_{p}^{2}$ = 0.018; Horizontal: *F* (1, 28) = 0.340, *p* = 565, ${\eta }_{p}^{2}$ = 0.013, Fig. 2). For daily motor function, no significant between-group differences were found in Amount of use (*F* (1, 28) = 0.080, *p* = 0.903, ${\eta }_{p}^{2}$ = 0.001) and Quality of use (*F* (1, 28) = 0.496, *p* = 0.599, ${\eta }_{p}^{2}$ = 0.010) on the PMAL-R (Fig. 2). However, compared to the therapist-based CIMT group, the kinect-based CIMT group exhibited greater nED during the PPV_before_ phase (*F* (1, 28) = 4.862, *p* = 0.036, ${\eta }_{p}^{2}$ = 0.158) and smaller nTD during the PPV_after_ phase (*F* (1, 28) = 6.021, *p* = 0.021, ${\eta }_{p}^{2}$= 0.188), as well as greater TCS in both phases (PPV_before_: *F* (1, 28) = 5.043, *p* = 0.033, ${\eta }_{p}^{2}$ = 0.162; PPV_after_: *F* (1, 28) = 6.283, *p* = 0.019, ${\eta }_{p}^{2}$ = 0.195, Fig. 3). No significant differences were found in the between-group comparisons of nED during the PPV_after_ phase (*F* (1, 28) = 0.141, *p* = 0.710, ${\eta }_{p}^{2}$ = 0.005) and nTD during the PPV_before_ phase (*F* (1, 28) = 1.091, *p* = 0.306, ${\eta }_{p}^{2}$ = 0.040, Fig. 3).

## Discussion

This study compared the effects of kinect-based CIMT and that of the therapist-based CIMT on UE and trunk motor control and on daily motor function in children with UCP. Compared to therapist-based CIMT, kinect-based CIMT yielded comparable improvement on UE motor control and daily motor function. Moreover, kinect-based CIMT demonstrated extra benefits on improving trunk motor control.

Kinect-based CIMT resolved the practical issues with CIMT, which improved the clinical utility of CIMT. To our knowledge, this kinect-based CIMT program is the first to integrate the CIMT principles into VR games. To restraint the less-affected UE, limb-specific games were designed for the Kinect sensor and a contextual restraint method was adopted. Contextual restraint was first adopted in camp-based CIMT [22]. In a Pirate-group CIMT, children with UCP were told to be pirates, and their less-affected UEs were restrained by slings due to injury. Restraint of the less-affected UE without negative emotion was successfully achieved in that study. A similar concept of restraint (i.e., contextual restraint) was extended and used in our study. Instead of physical restraint, our kinect-based CIMT program provides a playful restraint context wherein the players should put their less-affected hand on one knee to stabilize a box or to block a monster’s attacks. Moreover, the children with UCP were asked to collect cannonballs or catch fish and pick apples with only their affected UE. The children with UCP were involved in the playful contexts of the games with positive emotion and high engagement. These results indicated that the contextual restraint was effective even though the physical restraint was not applied. On the other hand, to prevent the problems derived from the intensive training protocol, repetitive intensive practice was embedded in the entertaining games and controlled by a computer. The results showed that children with UCP practiced a mean of 853 repetitions of targeted UE movements in each treatment session but still had high motivation and engagement. Implementing CIMT with a VR system may also reduce the burdens of families and therapists.

Kinect-based CIMT demonstrated effects comparable to that of therapist-based CIMT on the affected UE motor control and daily function. The possible factors which contributed to the motor improvement of the affected UE being similar to those of therapist-based CIMT were the intensive repetitive practice, structured training approach and functional motor training goals used in kinect-based CIMT. First, intensive repetitive practice, a crucial principle of CIMT, is embedded in the fun VR games of kinect-based CIMT. Intensive repetitive motor training can drive neuroplasticity and motor learning and thus improve the motor control of the affected UE [23]. Second, since a structured training approach is adopted in therapist-based CIMT, a therapist’s interface for adjusting the game difficulty levels was designed in the VR games of kinect-based CIMT. Similar to the therapists in therapist-based CIMT, therapists in kinect-based CIMT can adjust the difficulty of the game level to fit the child’s ability and progression. Providing just-right challenges also helped the motor learning of the affected UE [18]. Third, the training goals set for the affected UE in the VR games all focused on training the primary UE motor components of daily function [19], such as reaching and grasping. Although, unlike therapist-based CIMT, the kinect-based CIMT did not provide direct training in daily UE activities, practicing those critical UE movements also improved the daily motor function measured with the PMAL-R. The improved scores on the PMAL-R in both groups surpassed the values of minimal clinically important differences [24], indicating that improvements of daily function can be perceived by parents of children with UCP in daily life. The comparable effects on affected UE motor control and daily function of the kinect-based CIMT and therapist-based CIMT suggests that kinect-based CIMT can be considered as an alternative to therapist-based CIMT.

Compared with therapist-based CIMT, kinect-based CIMT may provide extra benefits on improving trunk motor control in children with UCP. During forward reaching, typically developing children tend to use their arm first, followed by the trunk if the target is too far to reach, which is an efficient strategy for forward reaching [25, 26]. In contrast, children with UCP usually use their trunk to compensate for the poor reaching function of their affected UEs [4]. In our study, compared to the therapist-based CIMT group, the children in the kinect-based CIMT group used greater arm movements during the early reaching (i.e., PPV_before_) phase and thus less trunk compensation during the late reaching (i.e., PPV_after_) phase after intervention. This difference indicated that after the 36-h intervention, the children in the kinect-based CIMT group were able to adopt an efficient reaching strategy similar to that of typically developing children. TCS results during both phases in between-group comparisons were also in agreement with findings on arm and trunk displacements, indicating that less trunk compensation was adopted by children with UCP in the kinect-based CIMT group after intervention. The improved trunk control of the children in the kinect-based CIMT group may be attributed to the pause design during gameplay, which was controlled by the kinect system. The pause mechanism in the kinect-based CIMT program was originally designed to prevent inadequate compensatory movements and ensure the children’s safety. It seems that this pause mechanism may provide an invisible restraint for children with UCP, resulting in improvement of trunk motor control during reaching. The effects on trunk motor control were also reported in previous studies which used harnesses to provide trunk restraint for children with UCP [4, 27]. Instead of using intrinsic tactile feedback provided by a harness, augmented extrinsic feedback provided by the pause mechanism was used in our study [28]. Using augmented extrinsic feedback to provide trunk restraint may eliminate the discomfort caused by long-term restraint by a harness, which may be an alternative approach to provide trunk restraint. The benefit of trunk restraint could be also considered in the application of CIMT.

During a pandemic (e.g., COVID-19), the kinect-based CIMT program may also serve as a no-contact or low-contact rehabilitation program. Due to COVID-19, social distancing (e.g., minimal to no contact) are imposed [29]. In our VR program, UE motor training for children with UCP can be implemented without therapist’s hands-on guides or oral cues, which helps reduce the risk of spreading diseases. Additionally, our kinect-based CIMT program could be further extended to telerehabilitation. As an approach to easy and safe access to health-care services, telerehabilitation that provides treatments and evaluations for clients has been increasing recently [30, 31]. Thus, in future studies, the effectiveness of the proposed kinect-based CIMT program delivered through telerehabilitation on the affected UE motor function of children with UCP could be investigated.

This study has some limitations. First, the sample size was relatively small. Moreover, the children with UCP in the present study encompassed a homogeneous population, so the generalizability of the study findings might be limited. Further research on larger and diverse samples is needed. Second, the long-term effects of the interventions were not investigated with follow-up assessments. Future studies on this topic are warranted. Third, the kinect-based CIMT program only consisted of two games. More games based on the CIMT principles can be developed in the future to improve the clinical utility of the kinect-based CIMT.

## Conclusion

The kinect-based CIMT has been developed and successfully resolves the issues with CIMT. In the present study, effects of the kinect-based CIMT and that of the therapist-based CIMT were compared. The results suggested that the kinect-based CIMT has effects comparable to that of therapist-based CIMT on UE motor control and daily motor function. Moreover, the kinect-based CIMT also has extra benefits on improving trunk motor control. Therefore, the kinect-based CIMT can be used as an alternative approach to the therapist-based CIMT, which would help to improve the clinical utility of CIMT.

## Data Availability

The data that support the findings of this study are available from the corresponding author upon reasonable request.

## Abbreviations

CIMT: Constraint-induced movement therapy
DD: Developmental disregard
MT: Movement time
MU: Movement unit
nED: Normalized endpoint displacement
nTD: Normalized trunk displacement
PMAL-R: Revised Pediatric Motor Activity Log
PPV: Percentage of movement time to peak velocity
PV: Peak velocity
RT: Reaction time
TCS: Trunk contribution slope
UCP: Unilateral cerebral palsy
UE: Upper extremity
VR: Virtual reality
